# Correlation analysis between eGFR_cys_ and SXscore in patients with diabetes

**DOI:** 10.3892/etm.2014.1536

**Published:** 2014-02-12

**Authors:** ZHONG YONG, LIYONG ZHU, JUAN TAN, SHAIHONG ZHU

**Affiliations:** The Third General Surgery of The Third Xiangya Hospital, Central South University, Changsha, Hunan 410006, P.R. China

**Keywords:** glomerular filtration rate, cystatin C, SYNTAX score, diabetes

## Abstract

The aim of the present study was to explore the association between the cystatin C-based estimated glomerular filtration rate (eGFR_cys_) and the SYNTAX score (SXscore) in patients with diabetes. To the best of our knowledge, this correlation has not been reported previously. The eGFR_cys_ and SXscore from 612 consecutive patients with diabetes were retrospectively included in this study. The patients were angiographically diagnosed with coronary artery disease (CAD) between July 2010 and March 2012 at the Department of Endocrinology. The SXscore was calculated using a previously described SXscore algorithm. Pearson correlations were used to analyze the correlation between eGFR_cys_ and SXscore. Patients with renal dysfunction were older, more often female and more likely to have a history of hypertension when compared with those with normal renal function. The eGFR_cys_ values were significantly lower and the cystatin C levels were significantly higher in the highest SXscore group than those in other groups (P<0.001). Correlation analysis indicated that eGFR_cys_ was negatively correlated with the SXscore (r=−0.7918, P<0.001). In addition, a significantly positive correlation was identified between levels of cystatin C and the SXscore (r=0.8891, P<0.001). In conclusion, eGFR_cys_ is an independent predictor of SXscore in patients with diabetes. The eGFR_cys_-estimating method may be considered important in the assessment of the SXscore in patients with diabetes.

## Introduction

Chronic kidney disease (CKD) has received increased focus in healthcare in recent years. There is a marked positive correlation between kidney dysfunction and cardiovascular disease (CVD) (1,2). Diabetes is recognized as a major risk factor for the development of CKD. CKD-associated diseases, including diabetes, have been proposed as coronary disease equivalents (3,4).

In certain kidney diseases, improving glomerular filtration rate (GFR) should be performed whenever serum creatinine (SCr) is measured. The most common method used to obtain an estimated GFR (eGFR) value is based on SCr. Early detection of kidney dysfunction in subjects with diabetes is of vital importance, as appropriate interventions have been shown to slow the progression to end-stage renal disease (ESRD) and reduce the incidence of CVD (5). SCr concentration is affected not only by GFR but also by a number of factors that are independent of GFR, including age, ethnicity, muscle mass, gender, use of medication and catabolic state (6–8).

Serum cystatin C is a low molecular weight protein that functions as a cysteine protease inhibitor and has been proposed to be a reliable marker of GFR for certain diseases associated with CVD (9). Serum cystatin C is a more effective indicator of GFR than SCr (10–12). At present, a number of serum cystatin C-based equations have been identified and proposed as alternative filtration markers to SCr-based equations (13–17).

Clinically, the SYNTAX score (SXscore) is capable of accurately detecting the severity and complexity of coronary artery lesions (18,19). The SXscore may also predict major adverse cardiac events (MACEs) and periprocedural myocardial infarction (MI) in patients undergoing elective percutanous coronary intervention (PCI) (20–24). In addition, a novel coronary artery bypass graft (CABG) SYNTAX score was identified by Farooq *et al* (25), which was capable of calculating the SYNTAX score in patients with prior bypass surgery.

Although numerous studies have investigated the correlation between renal dysfunction and the severity of coronary artery disease (CAD), these studies evaluated renal function using GFRs based on SCr concentration (3,4,25,26). Furthermore, CAD severity was based on the number of stenotic coronary arteries. To the best of our knowledge, the correlation between the cystatin C-based estimated GFR (eGFR_cys_) and SXscore in patients with diabetes has not been previously reported. Therefore, the aim of the present study was to evaluate the association between eGFR_cys_ and SXscore, and to explore the applicable value of eGFR_cys_ in patients with diabetes.

## Materials and methods

### Subjects

A total of 612 patients with diabetes were recruited to the study between July 2010 and March 2012 from the Department of Endocrinology, The Third Xiangya Hospital of Central South University (Changsha, China). All patients underwent coronary angiography (CAG) and were angiographically diagnosed with CAD. Patients with the following diseases were excluded from the study: Congenital heart disease, valvular heart disease, congestive heart failure and cardiomyopathy. In addition, patients that had undergone surgical revascularization were excluded.

The study design was approved by the Ethics Committee of the Third Xiangya Hospital, and informed consent was provided by all subjects.

### Renal function assessment

Prior to CAG, blood samples were collected from the antecubital vein while the patients were resting in the supine position. The serum concentration of cystatin C was measured using the particle-enhanced immunonephelometric method. eGFR_cys_ was calculated using the Chronic Kidney Disease Epidemiology Collaboration (CKD-EPI) cystatin C formula, as follows: eGFR_cys_ (ml/min/1.73 m^2^) = [127.7 × cystatin C^−1.17^ (mg/l) × age^−0.13^ × 0.91 (if female) × 1.06 (if African-American)] (27,28).

The eGFR_cys_ values were used to classify the patients into four groups, as follows: Group 1 (eGFR_cys_ ≥90 ml/min/1.73 m^2^); group 2 (60≤ eGFR_cys_ <90ml/min/1.73m^2^); group 3 (30≤ eGFR_cys_ <60 ml/min/1.73m^2^) and group 4 (eGFR_cys_ <30 ml/min/1.73 m^2^).

### Detection of cardiovascular risk factors

Patients were fasted overnight prior to the blood samples being obtained. The levels of total cholesterol (TC), triglycerides (TG), low/high-density lipoprotein cholesterol (LDL/LDH), creatinine, hemoglobin A1c (HbA1c), fasting plasma glucose, C-reactive protein (CRP) and body mass index (BMI) were measured in everyday practice.

### Angiographic and SXscore analysis

From the baseline diagnostic angiogram, each coronary lesion producing a ≥50% diameter stenosis in vessels ≥1.5 mm was scored separately and summed together to provide the overall SXscore, which was calculated using the SXscore algorithm (18,19). All angiographic variables pertinent to the SXscore calculation were computed by two of three experienced cardiologists who were blinded to the study of the angiograms. In the case of a disagreement, the opinion of the third observer was obtained and the final decision was made by consensus. Occluded infarct-related arteries in patients with acute MI were scored as occlusions of <3 months duration. Patients with in-stent restenosis lesions were scored in the same manner to those with *de novo* lesions. Patients with an SXscore >0 were angiographically defined as exhibiting CAD.

### Statistical analysis

In the present study, all of the data were processed using SPSS software 20.0 (SPSS, Inc., Chicago, IL, USA). A two-sided P-value of <0.05 was considered to indicate a statistically significant difference. Intergroup differences of categorical variables were compared using χ^2^ tests. Pearson and partial correlation coefficients were calculated between the eGFR and SXscore with adjustment for age, gender and other traditional risk factors. The ordinal logistic regression was employed to control for multiple covariates when analyzing the association between eGFR_cys_ and SXscore.

## Results

The average age of the 612 patients included in this study was 64.8±9.7 years. The mean eGFR_cys_ was 69.7±13.4 ml/min/1.73m^2^ (range, 8.4–154.3 ml/min/1.73 m^2^). Only 12 (5.7%) patients had an eGFR_cys_ <30 ml/min/1.73m^2^. The SXscore ranged between 1.0 and 53 with a mean of 17.4±7.5. Patients with renal dysfunction were older, more often female and more likely to have a history of hypertension when compared with those patients with normal renal function (Table I).

By detecting a number of risk factors, it was found that age, gender, systolic blood pressure (SBP), diastolic blood pressure (DBP), fasting glucose, HbA1c, TC, LDL, HDL, TG, BMI, CRP and incidences of hypertension and hyperlipidemia were not different between one group and another (data not shown). However, from the data in Table II, it was observed that eGFR_cys_ values were significantly lower and the levels of cystatin C were significantly higher in the highest SXscore group than those in other groups (P<0.001). Therefore, in combination with the results of Table I, the correlation between cystatin C level, eGFR_cys_ and the SXscore (including SXlow, SXmid, and SXhigh) was analyzed.

**Figure 1:**
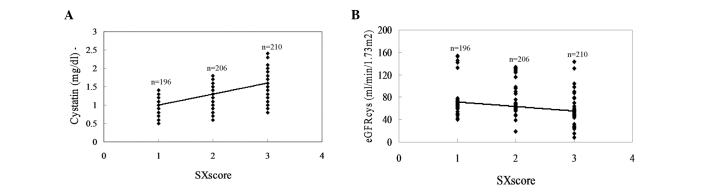
Correlation analysis of the cystatin C levels, eGFRcys and the SXscore in patients with diabetes. (A) Correlation between cystatin C and the SXscore; (B) correlation between the eGFR_cys_ and SXscore. ‘1’ represents the SXlow, ‘2’ represents the SXmid and ‘3’ represents the SXhigh. eGFR_cys_, cystatin C-based estimated glomerular filtration rate; SXscore, SYNTAX score;

The results of the correlation analysis indicated that there was a significant positive correlation between the levels of cystatin C and the SXscore (r=0.8891, P<0.001; Fig. 1A). In addition, the eGFR_cys_ was negatively correlated with the SXscore (r=−0.7918, P<0.001; Fig. 1B).

## Discussion

In the present study, it was demonstrated for the first time, to the best of our knowledge, that eGFR_cys_ was markedly associated with the SXscore in patients with diabetes. The results showed that renal function was correlated with the severity of CAD in patients with diabetes. This conclusion may be helpful to explain the increased risk of CVD-associated events and mortality in patients with renal dysfunction (26). Usually, diabetes is recognized as a major risk factor for the development of CKD. The cumulative incidence of MI may approach 20% over 10 years (4) when patients present with CKD and diabetes. Such effects of CKD and diabetes on mortality and MI have been extensively studied (29).

Clinically, the measurement of SCr concentration has been employed as an indirect method of estimating the renal function. The creatinine concentration has become the most commonly applied approximation of the GFR. However, this method has presented increasing limitations during clinical practice. Therefore, an improved method for the assessment of GFR is required. Serum cystatin C is freely filtered across the glomerular membrane, and is reabsorbed and completely metabolized in the proximal tubule. It has been demonstrated that serum cystatin C possesses a number of characteristics that make it an ideal endogenous GFR marker (6). Previous studies have shown that serum cystatin C is superior to serum creatinine as a GFR marker in patients with a mild or moderate reduction in GFR (11–13). Meta-analyses have also demonstrated that serum cystatin C is superior to creatinine in measuring renal function (10,30). A previous study indicated that all serum cystatin C-based equations, excluding the Larsson formula, were reliable markers of GFR in patients with CKD (31).

Clinically, the SXscore is a comprehensive angiographic scoring system that is capable of accurately detecting the severity and complexity of coronary artery lesions (18,19). The score was initially devised as a method to ensure that the cardiologist and the cardiac surgeon accurately reviewed the angiogram of patients with complex CAD, and it was important in stratifying patients with complex CAD to aid revascularization decisions (21). Further evaluation of the score has also indicated its ability to predict clinical outcomes in patients with three-vessel disease or unprotected left main disease, even the patients with CAD (22,23,32). The association between CKD and CAD has been investigated for a number of years. Kilickesmez *et al* (33) demonstrated that CKD has an independent influence on lesion morphology and complexity. Abaci *et al* (34) concluded that the eGFR was an independent predictor of the extension and severity of CAD among patients with diabetes. Kiyosue *et al* (35) revealed that the number of stenotic coronary arteries was higher in the CKD group compared with individuals without CKD. The results of the present study indicated that the correlation between eGFR_cys_ and the SXscore was significant, and that it may be an improved method of representing the correlation between kidney function and CAD severity.

In conclusion, this study demonstrated that eGFR_cys_ is an independent predictor of the SXscore in patients with diabetes. This may be beneficial in explaining the increased risk of CVD-associated events and mortality in patients with renal dysfunction. The eGFR_cys_-estimating method may be considered important in the assessment of the SXscore in patients with diabetes.

## Figures and Tables

**Table I:** Baseline data.

Characteristic	Group 1 (n=66)	Group 2 (n=242)	Group 3 (n=279)	Group 4 (n=25)	P-value
Age (years)	55.78±8.12	64.14±6.78	68.37±5.69	72.02±7.15	<0.001
Male (n)	57 (86.3)	191 (78.9)	125 (44.8)	7 (28.0)	<0.001
Hypertension (n)	37 (56.1)	181 (74.8)	224 (80.1)	23 (92.0)	<0.001
Hyperlipidemia (n)	35 (53.0)	119 (49.2)	127 (45.5)	11 (44.0)	0.544
Fasting glucose (mg/dl)	135.8±2.8	137.2±18.6	134.9±17.4	137.7±25.1	0.405
HbA1c (%)	7.7±2.5	7.4±2.7	7.5±1.2	7.6±1.8	0.624
TC (mg/dl)	228.5±21.4	231.4±23.2	235.6±17.3	237.6±22.5	0.082
LDL (mg/dl)	125.3±15.6	131.1±21.3	124.7±19.1	126.3±18.5	0.058
HDL (mg/dl)	54.5±11.4	55.1±16.3	53.4±15.6	57.1±11.5	0.083
TG (mg/dl)	112.9±21.7	113.7±20.3	116.8±11.9	118.6±22.3	0.205
BMI (kg/m^2)^	23.3±4.8	24.6±4.8	23.1±4.5	21.9±5.7	<0.001
CRP (mg/dl)	2.0±0.6	2.2±0.5	2.5±0.4	2.7±0.6	<0.001
Cr (mg/dl)	0.7±0.1	1.0±0.3	1.4±0.1	1.8±0.4	<0.001

Values in parentheses represent percentage. HbAlc, hemoglobin Alc; TC, total cholesterol; LDL, low-density lipoprotein cholesterol; HDL, high-density lipoprotein cholesterol; TG, triglyceride; BMI, body mass index; CRP, C-reactive protein; Cr, creatinine.

**Table II:** Comparison of parameters according to the SXscore.

Characteristic	SXlow (n=196)	SXmid (n=206)	SXhigh (n=210)	P-value
Cystatin C (mg/dl)	1.0±0.2	1.3±0.1	1.6±0.2	<0.001
eGFR_cys_ (ml/min/1.73m^2^)	69.9±20.8	62.0±19.4	54.3±22.5	<0.001
eGFR_cys_ (n)				<0.001
≥90	30 (15.3)	25 (12.1)	14 (6.6)	
≥60 and <90	91 (46.4)	90 (43.7)	65 (31.0)	
≥30 and <60	73 (37.2)	85 (41.3)	119 (56.7)	
<30	2 (2.1)	6 (2.9)	12 (5.7)	

Values in parentheses represent percentage. eGFR_cys_, cystatin C-based estimated glomerular filtration rate; SXscore, SYNTAX score. SXlow, SXscore ≤12.5; SXmid, SXscore >12.5 and ≤21.0; SXhigh, SXscore >21.0.
